# Postprandial effect of gastrointestinal hormones and gastric activity in patients with irritable bowel syndrome

**DOI:** 10.1038/s41598-023-36445-1

**Published:** 2023-06-09

**Authors:** Agata Furgała, Katarzyna Ciesielczyk, Magdalena Przybylska-Feluś, Konrad Jabłoński, Krzysztof Gil, Małgorzata Zwolińska-Wcisło

**Affiliations:** 1 Department of Pathophysiology, Faculty of Medicine, Jagiellonian University Medical College, Czysta 18 Str, 31-121 Kraków, Poland; 2 Department of Gastroenterology and Hepatology, Faculty of Medicine, Jagiellonian University Medical College, Kraków, Poland; 3 Department of Gastroenterology and Hepatology, Faculty of Medicine, Institute of Clinical Dietetics, Jagiellonian University Medical College, Kraków, Poland; 4 Department of Medical Education, Faculty of Medicine, Jagiellonian University Medical College, Kraków, Poland

**Keywords:** Gastroenterology, Gastrointestinal hormones

## Abstract

Altered gut regulation, including motor and secretory mechanisms, is characteristic of irritable bowel syndrome (IBS). The severity of postprandial symptoms in IBS patients is associated with discomfort and pain; gas-related symptoms such as bloating and abdominal distension; and abnormal colonic motility. The aim of this study was to assess the postprandial response, i.e., gut peptide secretion and gastric myoelectric activity, in patients with constipation-predominant IBS. The study was conducted on 42 IBS patients (14 males, 28 females, mean age 45.1 ± 15.3 years) and 42 healthy participants (16 males, 26 females, mean age 41.1 ± 8.7 years). The study assessed plasma gut peptide levels (gastrin, CCK—Cholecystokinin, VIP—Vasoactive Intestinal Peptide, ghrelin, insulin) and gastric myoelectric activity obtained from electrogastrography (EGG) in the preprandial and postprandial period (meal–oral nutritional supplement 300 kcal/300 ml). Mean preprandial gastrin and insulin levels were significantly elevated in IBS patients compared to the control group (gastrin: 72.27 ± 26.89 vs. 12.27 ± 4.91 pg/ml; p < 0.00001 and insulin: 15.31 ± 12.92 vs. 8.04 ± 3.21 IU/ml; p = 0.0001), while VIP and ghrelin levels were decreased in IBS patients (VIP: 6.69 ± 4.68 vs. 27.26 ± 21.51 ng/ml; p = 0.0001 and ghrelin: 176.01 ± 88.47 vs. 250.24 ± 84.55 pg/ml; p < 0.0001). A nonsignificant change in the CCK level was observed. IBS patients showed significant changes in postprandial hormone levels compared to the preprandial state—specifically, there were increases in gastrin (p = 0.000), CCK (p < 0.0001), VIP (p < 0.0001), ghrelin (p = 0.000) and insulin (p < 0.0001). Patients with IBS showed reduced preprandial and postprandial normogastria (59.8 ± 22.0 vs. 66.3 ± 20.2%) compared to control values (83.19 ± 16.7%; p < 0.0001 vs. 86.1 ± 9.4%; p < 0.0001). In response to the meal, we did not observe an increase in the percentage of normogastria or the average percentage slow-wave coupling (APSWC) in IBS patients. The postprandial to preprandial power ratio (PR) indicates alterations in gastric contractions; in controls, PR = 2.7, whereas in IBS patients, PR = 1.7, which was significantly lower (p = 0.00009). This ratio reflects a decrease in gastric contractility. Disturbances in the postprandial concentration of gut peptides (gastrin, insulin and ghrelin) in plasma may contribute to abnormal gastric function and consequently intestinal motility, which are manifested in the intensification of clinical symptoms, such as visceral hypersensitivity or irregular bowel movements in IBS patients.

## Introduction

Patients with irritable bowel syndrome (IBS) often experience intestinal symptoms after eating a meal, abdominal pain or discomfort associated with changing bowel habits^1,2^. Due to predominant symptoms, IBS is divided into four groups: IBS-C, with predominant constipation; IBS-D, with predominant diarrhea; IBS-M, with mixed bowel habits; and IBS-U, in which the predominant symptoms are unclassified^3^.

The diagnosis of IBS relies on Rome IV Diagnostic Criteria for Disorders of Gut-Brain Interaction (DGBI) with a timeframe of recurrent abdominal pain (on average at least one day/week in the last three months, occurring at least 6 months prior to diagnosis) associated with defecation, a change in the frequency of stool and changes in the appearance of stool (the Bristol stool form scale—BSFS)^3^.

Postprandial symptoms show a prolonged and exaggerated gastroenteric and gastrocolonic response, mediated by gastrointestinal hormonal stimulation of colonic smooth muscle or by neural reflexes originating from the upper part of the gastrointestinal tract^4^. Peristalsis is under myogenic control, hormonal control, and neural control and can be activated by chemical or mechanical stimuli detected by enteroendocrine cells and mechanosensitive neurons in the enteric ganglia. Interstitial cells play a role in transmitting excitatory-inhibitory signals between the enteric nervous system and smooth muscle cells^5^.

Normal gastrointestinal motility results from coordinated smooth muscle contractions derived from two basic patterns of electrical activity in smooth muscle cell membranes, i.e., slow waves and spike potentials. Slow-wave activity is a property of smooth muscles independent of nerve stimuli. Importantly, slow waves are not action potentials and do not elicit contractions themselves. Instead, they coordinate or synchronize muscle contractions in the gut by controlling the appearance of the second type of depolarization—i.e., spike potentials—which occurs only at the crests of slow waves. The strength of the contraction depends on the amplitude and number of action potentials. The factors inducing action potentials are stretching, nerve impulses of the enteric nervous system and the release of digestive hormones such as ghrelin, gastrin, cholecystokinin, and motilin^6^.

Clinically, the gastrocolic reflex is associated with the pathogenesis of irritable bowel syndrome: eating or drinking may cause an excessive gastrocolic response due to their associated increased visceral sensitivity, which may lead to abdominal pain, diarrhea, or constipation. Studies have previously reported that IBS patients show alterations in their autonomic, immunologic and visceral responses to food intake^7,8^. After a meal, IBS patients may experience a strong urge to defecate and symptoms such as abdominal bloating, flatulence, pain, tenesmus, and relief of symptoms following defecation. The reflex is coordinated by the autonomic nervous system, the enteric nervous system and the cells of the digestive tract that regulate endocrine functions. Impairment of nervous or hormonal mechanisms leads to the reduction or elimination of gastroesophageal reflux, which slows the passage of feces through the intestines and colon, leading to functional constipation^9^. In addition, the impact on the gut microbiome may have a downstream effect that alters the ability of enteroendocrine cells to detect and perform paracrine functions, thereby indirectly affecting colon motility^10^.

It is known that the hormones of the brain–gut axis regulate gut motility. Motilin, cholecystokinin (CCK), glucose-dependent insulinotropic peptide (GIP), glucagon-like peptide-1 (GLP-1), peptide YY (PYY) and ghrelin are major gut hormones. In IBS patients, changes in intestinal hormone secretion are observed, both in the fasting state and postprandially^11^. Although the role of enterohormones in gastrointestinal disease seems to be well established, many important questions still need to be answered. In these studies, meals were a way to induce gastrointestinal symptoms in IBS patients, which we recorded by examining the gastric myoelectrical activity and hormonal secretion (gastrin, CCK, vasoactive intestinal peptide (VIP), ghrelin, insulin) response and then comparing the changes in IBS patients to those of healthy controls. Understanding these relationships can be useful for developing new IBS diagnostic techniques and considering new treatment options.

## Materials and methods

### Investigated subjects

#### Clinical procedure

The study included 84 participants, who were divided into two groups:

##### IBS group

This group included 42 patients with constipation-predominant IBS (14 males, 28 females, mean age 45.1 ± 15.3 years). The mean duration of IBS was 5 ± 1.5 years. Qualified IBS patients were diagnosed according to the Rome IV Diagnostic Criteria for DGBI and recruited after examination by gastroenterologists from the Gastroenterology and Hepatology Department Medical College Jagiellonian University in Cracow.

##### Control group

This group included 42 healthy volunteers (16 men, 26 women), matched with the IBS group by age and sex (41.1 ± 8.7 years), with no history of GI disorders. Healthy controls were recruited from the University Hospital's employees by means of advertisements (online and on bulletin boards). Every volunteer completed a health questionnaire and was examined by a physician.

All participants were interviewed to obtain a detailed medical history. The clinical characteristics of the enrolled participants are presented in Table 1.

**Table 1:** Characteristics of IBS patients and healthy controls.

Parameters	Control group	IBS patients	*p*
*N*	42	42	
Age [years]	43.1 ± 12.7	45.1 ± 15.1	NS
Sex (f/m)	26/16	28/14	NS
BMI [kg/m^2^]	24.7 ± 2.24	25.6 ± 2.47	NS
HR (beat/min)	65.2 ± 9.21	67.9 ± 10.8	NS
BPs [mmHg]	125.2 ± 9.2	123.4 ± 7.4	NS
BPd [mmHg]	84.2 ± 8	80.6 ± 9.1	NS
IBS duration (years)	–	5 ± 1.5 (2–18)	–
CRP [ug/ml]	1.5 ± 1.8	3.36 ± 1.7	**0.002**
IFN-α [pg/ml]	1.43 ± 0.9	1.22 ± 1.27	NS

*IBS* irritable bowel syndrome, *BMI* body mass index, *HR* heart rate, *BPs* systolic blood pressure, *BPd* diastolic blood pressure, *CRP* C-reactive protein, *IFN*-*α* interferon alpha,

*p* statistically significant differences between groups: IBS and control (p < 0.05), *NS* nonsignificant. Significant values are in bold.

The exclusion criteria were as follows: diabetes mellitus; obesity (BMI ≥ 30 kg/m^2^); tobacco smoking; alcohol abuse; cardiovascular diseases (e.g., hypertension, coronary artery disease, and valvular heart disease); gastrointestinal pathology (e.g., inflammatory bowel disease); gastrointestinal surgery; renal or gynecological pathology that might result in IBS symptoms (e.g., bowel resection, endometriosis); current medications taken for IBS regularly (e.g., antidiarrheals, laxatives, antispasmodics, or 5-HT3 antagonists three or more times a week); the intake of medications known to interfere with gastric myoelectric activity and autonomic function; and a history of chronic diseases.

All participants were asked to fast for at least 12 h before the tests and refrain from taking medications known to affect autonomic function and GI motility during the three days of the study.

### Methods

#### Assessment of gastric myoelectric activity—electrogastrography (EGG)

Thirty-minute recordings of gastric myoelectric activity (GMA) under basal conditions were obtained in both groups after an overnight fast and one hour after a standard liquid meal (oral nutritional supplement—ONS: Nutridrink, Nutricia  Poland, 300 kcal/300 ml; 16% proteins (9.6 g/100 ml), 49% carbohydrates (29.7 g/100 ml) and 35% fat (9.3 g/100 ml)). EGG was performed using four-channel electrogastrography Polygraf NET (Medtronic, USA). The following EGG parameters were evaluated: the percentage of normogastria time (2–4 cycles per minute (cpm)), the percentage of bradygastria time (1.0–2 cpm); the percentage of tachygastria time (4–10 cpm), the percentage of dysrhythmia time (not classified as bradygastria or tachygastria slow wave frequency); the period dominant frequency (PDF), the period dominant power (PDP) of the dominant frequency (as the power or amplitude of the DF) and the average percentage of slow-wave coupling (APSWC) defined as the percentage time within a given period during which the difference in DF between two channels is lower than 0.2 cpm, i.e., the rate of coupling between individual channels allowing for the identification of isolated disturbances along the gastric slow-wave path—an averaged index of coupling pertaining to six possible channel pairs^12–14^.

Changes in the EGG dominant power likely reflect gastric contractility^15,16^. Gastric myoelectric activity includes slow waves (referred to as electrical control activity) and spike potentials associated with electrical response activity^14,17^. The frequency of standard gastric slow waves (normogastria) in humans corresponds to approximately 3 cycles per minute, with a range of 2–4 cmp, which occurs > 70% of the time. Potential deviations from normogastria include gastric dysrhythmias (bradygastria, tachygastria, and arrhythmia), electromechanical uncoupling and abnormal slow-wave propagation.

EGG is a noninvasive technique for recording gastric electrical activity (GEA) from the abdominal surface, which indicates a cause and effect relationship between gastric motility abnormalities and dyspeptic symptoms^18^. GEA consists of spontaneous rhythmic electrical activity that determines the timing and frequency of contractile activity, including gastric emptying^19^. EEG has some limitations in representing stomach contractions, their shape, pattern, and frequency. Recording with skin electrodes is subject to numerous movement artifacts and electrical interferences from other organs. EGG parameters were compared with parameters obtained from other techniques of gastrointestinal motility measurement, so simultaneous recordings by cutaneous and internal (serosal and mucosal) EGG have yielded similar findings^20,21^. A study on canines demonstrated that while each gastric slow wave is accompanied by a contraction, the latter disappears in the case of dysrhythmia; furthermore, a relative increase in EGG PDP has been shown to be associated with an increase in gastric contractile activity^22^. Therefore, we can use EGG as a reliable measurement method, recording gastric slow waves and parameters derived from spectral analysis, providing clinically relevant information about gastric motility. The technical limitation of EGG is the relatively low signal-to-noise ratio of the signal, and the applicability and reproducibility of the method have depended on technological developments, appropriate equipment settings, the development of new amplifiers and signal quality.

#### Biochemical assays

Blood samples for determination of plasma concentration of CCK, VIP, gastrin, insulin and ghrelin were simultaneously collected at 8:00 a.m., shortly before recording the EGG (at rest in a preprandial state) and one hour after a standard meal (postprandial state). Blood samples were collected in tubes containing potassium EDTA (50 μl liquid/ml blood). Blood samples were stored on ice until centrifugation (3800*g *at 8 °C for 10 min) to separate the plasma as soon as possible after being obtained. The supernatant was aspirated and stored until analysis (6 h to 1 month at − 20 °C). The determinations were performed using a double-antibody sandwich enzyme-linked immunosorbent assay (ELISA) in the Biochemical Laboratory.

In the laboratory assessment, the following kits were used for the individual test: gastrin—ELISA kit Gastrin I (G17), R & D Systems Inc.—USA (sensitivity 0.7 pg/ml); cholecystokinin—ELISA kit Bender MedSystems Austria (sensitivity 1.65 ng/ml); ghrelin—ELISA kit Demeditec Diagnostic GmbH, Germany (sensitivity 25 pg/ml); Vasoactive Intestinal Peptide (VIP)—ELISA kit Phoenix Pharmaceuticals Inc, USA (sensitivity 0.13 ng/mL); insulin—INS-EASIA kit BioSource, Europa S.A. Belgium (KAP 1251).

#### Bioethics

The study was conducted in accordance with the Helsinki Declaration, and the study protocol was approved by the Local Bioethics Committee at Jagiellonian University in Krakow (permission no. 1072.6120.173.2018). All participants gave their written informed consent to participate in the study.

#### Statistical analysis

Analyzing the data distribution in the studied groups to avoid the required normal distribution, we decided to apply a distribution-free test suitable for a small population. Differences in paired data were analyzed with Wilcoxon’s test, and differences in unpaired data were analyzed with the Mann–Whitney test. Depending on the distribution type, the significance of intragroup differences was verified with two-way ANOVA followed by the Kruskal–Wallis post hoc test, which was performed to examine the differences between patients and the control group when data normality was not present. The association between enterohormone plasma levels and EGG was evaluated by analyzing the relationship between plasma levels and EGG parameters (the percentage of normogastria, tachygastria, bradygastria, and dysrhythmias, PDF, PDP, and APSWC). The correlation between analyzed data was determined with Spearman’s test. TIBCO Statistica for Windows, version 13.3 PL (TIBCO Software Inc., Palo Alto, CA, USA, Jagiellonian University license), software was used for statistical analyses. Data are presented as the mean ± SD, median (Me) and min–max values. The threshold of statistical significance for all the tests was set at p < 0.05.

### Results

Eighty-four participants were enrolled in the study: 42 patients with IBS (28 female, 14 male) and 42 healthy volunteers (26 female, 16 male). The median disease duration for IBS patients (defined as Roma IV Diagnostic Criteria of DGBI) was 5 ± 1.5 years. Although the CRP levels in IBS patients were within the normal range, IBS patients showed higher CRP levels than healthy volunteers (3.36 ± 1.7 µg/ml vs. 1.5 ± 1.8 µg/ml, p = 0.002). This indicates that patients had low-grade inflammation (Table 2).

**Table 2:** Enterohormone levels in IBS patients compared with the control group in the preprandial and postprandial periods.

Peptides	Preprandial period					Postprandial period					P*- hormone preprandial vs. postprandial				
Mean ± SD Me (min–max)	Gastrin [pg/ml]	CCK [ng/ml]	VIP [ng/ml]	Ghrelin [pg/ml]	Insulin [IU/ml]	Gastrin [pg/ml]	CCK [ng/ml]	VIP [ng/ml]	Ghrelin [pg/ml]	Insulin [IU/ml]	*p* Gastrin	*p* CCK	*p* VIP	*p* Ghrelin	*P* Insulin
Control group (n = 42)	12.27 ± 4.92 13.55 (4.2–28.8)	2.98 ± 1.6 2.31 (0.87–7.41)	25.96 ± 4.61 25.61 (16.41–40.59)	256.05 ± 83.17 243.04 (123.8–547)	8.04 ± 3.21 7.1 (4.5–19.3)	16.23 ± 6.53 15.4 (4.8–34.2)	3.51 ± 1.59 3.15 (0.98–7.98)	27.42 ± 6.67 26.88 (15.34–51.07)	197.76 ± 72.81 181.32 (101–517)	25.58 ± 11.65 23.00 (11.4–59.7)	** < 0.00001**	**0.0008**	**0.02**	** < 0.00001**	** < 0.00001**
IBS patient group (n = 42)	72.27 ± 26.89 78.20 (17.8–123.4)	0.21 ± 0.13 0.19 (0.04–0.65)	6.69 ± 4.78 4.35 (0.36–15.5)	180.05 ± 93.22 165.75 (22.45–370)	15.31 ± 12.92 11.25 (4.2–75.0)	102.91 ± 27.83 100.65 (22.1–156.1)	0.94 ± 0.74 0.95 (0.12–3.2)	10.10 ± 5.34 7.45 (3.4–21.2)	155.59 ± 82.24 141.65 (14.9–342.1)	64.46 ± 24.29 29.7 (8.9–138.9)	** < 0.00001**	** < 0.00001**	** < 0.00001**	** < 0.00001**	** < 0.00001**
*P*^*#*^*—*control vs. IBS	** < 0.00001**	** < 0.00001**	** < 0.00001**	**0.0001**	**0.0001**	** < 0.00001**	** < 0.00001**	** < 0.00001**	**0.003**	0.086					

P*—statistically significant response to meals in IBS patients and healthy controls (Wilcoxon signed rank test); P^#^—statistically significant differences between the healthy controls and IBS patients before and after meals (Mann–Whitney U test). Significant values are in bold.

*Me* median.

### Biochemical assays

#### Plasma concentrations of enterohormones

Irritable bowel syndrome patients presented a higher median fasting concentration of gastrin than healthy controls, 78.30 pg/ml vs. 13.55 pg/ml (p < 0.00001), which significantly increased after the meal in both groups to 100.65 pg/ml (p < 0.00001) vs. 15.40 pg/ml (p < 0.00001), respectively. The postprandial plasma concentration of gastrin was still higher in the group of patients than in the control group (p < 0.00001) (Table 2).

In patients with IBS, preprandial CCK levels were significantly lower (0.19 ng/ml) than those in the control group (2.31 ng/ml; p < 0.00001), with a significant increase in response to the standard meal to 0.95 ng/ml (p < 0.00001) vs. 3.15 ng/ml (p = 0.0008) in both groups, respectively. After the meal, CCK plasma concentrations were lower in IBS patients than in healthy controls (p < 0.000010) (Table 2).

In the fasting period, the plasma concentrations of VIP peptide were lower in patients with IBS (4.35 ng/ml) than in the control group (25.61 ng/ml; p < 0.00001), which increased after the meal to 7.45 ng/ml (p < 0.00001) vs. 26.88 ng/ml (p = 0.02) in both groups. The plasma VIP levels after the meal were as before lower in the IBS group than in the control group (p < 0.00001) (Table 2).

The median fasting plasma concentrations of ghrelin in IBS patients were significantly lower than those in healthy controls (165.75 vs. 243.04 pg/ml; p = 0.0001). After the meal, there was a significant decrease in both the IBS patient and control groups (141.65; p < 0.00001 vs. 181.31 pg/ml, p < 0.00001). Postprandial ghrelin concentrations were lower in patients than in controls (p = 0.0027) (Table 2).

Fasting insulin levels were substantially higher in IBS patients (11.25 vs*.* 7.1 μIU/ml; p = 0.0001). Postprandial insulin levels increased significantly in both the IBS patient and control groups (29.7; p < 0.00001 vs*.* 23.0 μIU/ml; p < 0.00001), but there were no statistically significant differences between groups (p = 0.087). The detailed results of the data are presented in Table 2.

### Electrogastrography

#### EGG preprandial period

During examinations in a preprandial state, patients with IBS presented significantly lower percentages of normogastria time (59.8 ± 22.0 vs. 83.19 ± 16.7; p < 0.00001) and APSWC (58.26 ± 15.0 vs. 72.66 ± 16.0 %; p < 0.00001) than the control group. In IBS patients, preprandial analysis showed dysrhythmia (arrythmia) during 25.51 ± 15.3 % of the recording time compared to 8.71 ± 8.2 % in the control group (p < 0.05). In addition, nonsignificantly higher values of period dominant power were observed (PDP: 94,4661 ± 23,728 [μV^2^] vs. 92,157 ± 17,468 [μV^2^]; p = 0.92), which did not increase significantly after the meal in the IBS patient group.

#### EGG response to food—postprandial

**Figure 1:**
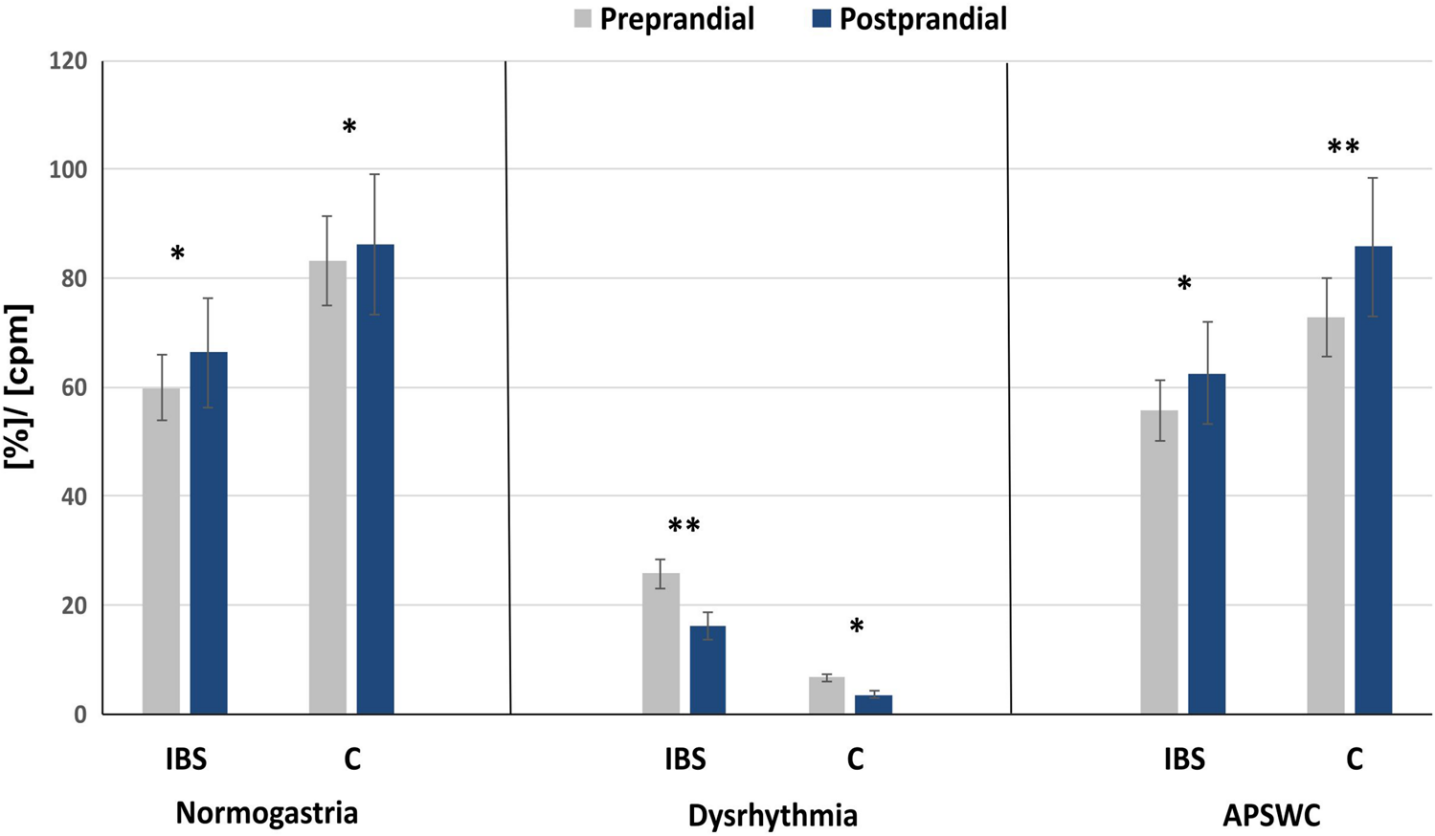
Comparison of the significant differences in response to feeding in gastric myoelectric activity in IBS patients and the control group. *APSWC* average percentage slow-wave coupling, *IBS* IBS patients, *C* the control group; *p = 0.04; **p = 0.002—Wilcoxon signed-rank test.

Abnormal EGG response to the meal was defined as no increase in dominant power^23^. A postprandial to preprandial power ratio (PR) < 1 indicates a decreased distal gastric motor response to meals. All healthy controls showed a normal response to the meal. Abnormal PR responses were documented in 15 (34.8%) IBS patients. Changes in EGG parameters recorded after eating the standard meal are summarized and presented in Table 3 and Fig. 1.

**Table 3:** Parameters of gastric myoelectric activity obtained from multichannel electrogastrography in patients with IBS and the control group.

EGG	Parameters Mean ± SD Median (Min–max)	Control group (n = 42)	IBS patients group (n = 42)	p U Mann–Whitney test
**Preprandial period**	PDF [cpm]	2.97 ± 0.3 2.95 (2.1–3.8)	2.93 ± 0.7 2.9 (0.6–4.8)	0.75
	PDP [μV^2^]	92,157 ± 17,468 49,773 (101–523,328)	944,661 ± 23,728 25,469 (850–640,887)	0.92
	Normogastria [%]	83.19 ± 16.7 85.26 (13.3–100.0)	59.8 ± 22.0 54.50 (19.4–100.0)	** < 0.00001**
	Bradygastria [%]	2.26 ± 4.0 0 (0–17.6)	7.67 ± 6.7 6.5 (0–25)	** < 0.00006**
	Tachygastria [%]	3.72 ± 4.9 2.5 (0–21.9)	5.91 ± 6.4 3.8 (0–29)	0.08
	Dysrhythmia [%]	8.71 ± 8.2 6.8 (0–31)	25.51 ± 15.3 25.8 (0–62.1)	** < 0.00001**
	APSWC [%]	72.66 ± 16.0 72.85 (13.39–100)	58.26 ± 15.0 55.60 (13.55–94.8)	** < 0.00001**
**Postprandial period**	PDF [cpm]	3.1 ± 0.3 3.05 (2.6–3.8)	3.0 ± 0.5 2.95 (2.1–4.8)	0.054
	PDP [μV^2^]	145,705 ± 150,469 83,352 (10,635–613,422)	92,627 ± 164,120 43,500 (3200–985,898)	0.135
	Normogastria [%]	86.1 ± 9 85.5 (72.3–100.0)*	66.3 ± 20.2 62.2 (31–100.0)*	** < 0.00001**
	Bradygastria [%]	2.3 ± 3.7 0 (0–15.4)	7.6 ± 7.3 5.9 (0–25.8)	**0.00007**
	Tachygastria [%]	5.6 ± 4.5 3.5 (0–12.1)	8.6 ± 7.4 6.7 (0–25.8)	**0.033**
	Dysrhythmia [%]	5.9 ± 6.1 3.6 (0–18.2)*	17.6 ± 11.7 16.2 (0–41.4)*	** < 0.00001**
	APSWC [%]	82.8 ± 10.9 85.7 (55–98.4)*	63.8 ± 13.2 62.5 (42.4–92.9)*	** < 0.00001**
	Postprandial to preprandial power (PR) ratio	2.7 ± 1.7 1.8 (1.1–7.3)	1.7 ± 1.6 1.3 (0.8–7.1)	**0.00009**

*PDF* period dominant frequency, *PDP* period dominant power, *APSWC* average percentage slow-wave coupling.

*Significant differences in response to the meal (Wilcoxon signed rank test). Significant values are in bold.

A reduction in dysrhythmia was observed after a meal to 17.6 ± 11.7 % and 5.9 ± 6.1 % in the IBS patients and healthy controls, respectively. The average slow-wave coupling percentage was lower in the preprandial period and increased in IBS patients and the control group to 62.46 ± 12.63 and 82.65 ± 10.78 %, respectively. In contrast to the controls, IBS patients showed no improvement in the percentage time of bradygastria, PDP, or PDF during examinations in a postprandial state (p<0.05). The PDP in the control group increased almost fold from 92,157 ± 17,468 [μV^2^] to 145,705 ± 150,469 [μV^2^], p = 0.04.

Abnormal EGG is defined as less than 70% of the recording time as 2–4 cpm slow waves^23,24^. None of the healthy controls showed EGG abnormalities in either the preprandial or postprandial state. In contrast, abnormal EGG recordings were obtained for 25 (59.5%) IBS patients examined in a preprandial state and 22 (52.3%) IBS patients examined in a postprandial state. Overall, EGG abnormalities were found in 19 study participants (45.23%).

### Correlation analysis of the parameters tested in the IBS group: enterohormone plasma levels with BMI, body weight, sex, age and EGG parameters

We analyzed associations between the plasma concentrations of enterohormones and BMI, body weight, sex and age. The data showed no relationship between plasma levels of CCK, VIP, gastrin, insulin and ghrelin and any of these variables.

The analysis of the association between plasma levels of CCK, VIP, gastrin, insulin and ghrelin and EGG parameters in the preprandial and postprandial states showed the following:

1. In the preprandial state, the data did not show a relationship between plasma levels of CCK, VIP, gastrin, insulin and ghrelin and EGG parameters.
2. In the postprandial state, insulin levels were negatively correlated with the percentage of bradygastria time (R = − 0.323; p = 0.042). The postprandial gastrin plasma concentration was negatively correlated with PDP (R = − 0.324; p = 0.035), the percentage of normogastria time (R = − 0.331; p = 0.035) and APSWC (R = − 0.347; p = 0.024) (Table 4).
3. The correlation analysis of the percentage changes in preprandial to postprandial parameters (EGG, enterohormones) showed that ghrelin was negatively correlated with normogastria in IBS patients (R = − 0.338; p = 0.028).

**Table 4:** The correlation between plasma levels of enterohormones and EGG parameters during the preprandial and postprandial states.

Parameters of correlation	R	P value
EGG vs. enterohormones—postprandial state		
Insulin vs. bradygastria	− 0.323	0.042
Gastrin vs. PDP	− 0.324	0.035
Gastrin vs. normogastria	− 0.331	0.035
Gastrin vs. APSWC	− 0.347	0.024
∆ of EGG vs. ∆ of enterohormones		
Ghrelin vs. normogastria	− 0.338	0.028

Comparison between the PR ratio of EGG and the post/preprandial ratio of gut peptides.

*PDP* period dominant power, *APSWC* average percentage slow-wave coupling, *∆* the percent changes of pre- to postprandial parameters, *R* Spearman correlation coefficient.

## Discussion

These studies were undertaken to better understand the disturbed release of selected intestinal hormones and gastric myoelectric activity that may be associated with eating and digesting food in patients with IBS. The results indicate that abnormalities in these parameters may contribute to intestinal motility dysfunction and disease symptoms. In patients with IBS-C, postprandial exacerbations of symptoms, as well as adverse reactions to certain types of foods, are common^25^. We examined the levels of interstitial peptides released from the stomach and upper small intestine under fasting and postprandial conditions and correlated them with gastric myoelectric activity in IBS patients.

In our study, we showed significantly elevated CRP levels in IBS patients compared to healthy controls, but these levels were still within the normal range, which can be a sign of low-grade inflammation. Microinflammation is considered to be a part of the pathogenesis of IBS^26,27^. Low-grade inflammation may affect the clinical picture through increased visceral hypersensitivity and altered bowel motility. IBS patients are genetically more prone to the development of inflammation due to high expression of TNF-a and low secretion of IL-10, which results in dysfunction of the colonic epithelial barrier^28,29^. Elevated levels of TNF-α and inflammatory mediators may be sufficient to sensitize sensory nerve endings, affect the excitability and sensory threshold of neurons and cause visceral hypersensitivity by increasing neuronal calcium influx and increasing the firing rate of submucosal neurons^30^.

In our investigation, we focused on differences in fasting and postprandial plasma intestinal peptide concentration levels in IBS patients compared to control subjects, which correlated with disturbances in gastric myoelectric activity.

*Gastrin* controls acid secretion in the postprandial period and regulates parietal cell maturation, enterochromaffin-like (ECL) cell numbers and histamine production capacity. In our study, fasting serum gastrin levels were significantly higher in patients with IBS than in healthy controls. After the meal, the serum gastrin level increased in both the control and IBS groups. However, postprandial gastrin levels are higher in IBS than the already very high basal fasting gastrin levels. An increase in gastrin and a decrease in somatostatin in all IBS subtypes may result in high levels of gastric acid secretion, which may explain the frequent occurrence of dyspepsia and gastroesophageal reflux in IBS patients^31,32^. Motor activity of the large intestine correlates with the increase in gastrin concentrations after a meal, and it has been suggested that gastrin plays a role in the gastrocolic response. It has also been suggested that the symptoms of abdominal pain in many IBS patients may be caused by an abnormal response to the concentration of circulating gastrin^33^.

EGG is a noninvasive method for the measurement of gastric myoelectrical activity that uses abdominal surface electrodes^34^. While direct techniques examined gastric motility (by measuring gastric emptying time) can only be used to determine the postprandial motor status, the EGG accurately determines indirectly gastric motor function in both the preprandial and postprandial states^34,35^. In published studies the positive predictive value of EGG for normal gastric emptying ranges from 65 to 100% (average 85%) and from 50 to 81% for predicting abnormal gastric emptying (average 65%)^13,36^. An increase in the percentage of normal slow waves, period dominant frequency and period dominant power and the accompanying reduction in dysrhythmia (arrhythmia) parameters is usually observed in healthy subjects as a gastric myoelectric response to food^13,14^.

Our EGG analysis detected a decreased power ratio in IBS patients because of reduced gastric distension or contractility and slower gastric emptying. Delayed gastric emptying has been shown to be associated with EGG changes, namely, a lower percentage of normal gastric slow waves and a lower postprandial increase in EGG period dominant power^13^. These results are consistent with those of other previous studies^37,38^. Although this delay is not always clinically apparent, the range of gastrointestinal symptoms may include early satiety, nausea, fullness, and bloating^39^. Inadequate gastric emptying in IBS patients may be associated with decreased vagal nerve discharge, and secondary to this, we observed an increase in ghrelin plasma concentration before a meal. In our previous studies, gastric myoelectric dysfunction, as manifested by abnormal gastric rhythm resulting in delayed gastric emptying and dyspeptic symptoms in IBS patients, was confirmed by ANS (the autonomic nervous system) dysfunction in an analysis of heart rate variability (HRV) and catecholamine levels^40^. Patients with constipation-related bowel dysfunction exhibit autonomic dysfunction, decreased vagal nerve activity, and increased sympathetic activity^41^.

Collectively, our results suggest that IBS constipation patients experience reductions in the gastric emptying rate and elevated plasma gastrin levels. Increased gastrin release may manifest a compensatory mechanism for decreased gastric myoelectrical activity. Additionally, the increase in gastrin concentration after a meal was correlated with low PDP, APSWC and normogastria, which are indicators of gastrointestinal dysmotility.

*Vasoactive intestinal peptide* is crucial for gastrointestinal tract homeostasis by maintaining the intestinal epithelial barrier and acts as a potent anti-inflammatory mediator that contributes to intestinal bacterial tolerance. As an anti-inflammatory and immunomodulatory agent, VIP inhibits the proinflammatory cytokines TNFα, IL-6, and IL-12 and promotes the production of the anti-inflammatory cytokine IL-10^42^. The VIP receptor occurs in lymphocytes, monocytes, macrophages, mast cells, microglia, and dendritic cells^43,44^. VIP levels were significantly lower in IBS patients than in healthy controls in our study, possibly due to constipation, which is the main symptom in our study group.

Research on idiopathic chronic constipation demonstrated decreased concentrations of VIP and abnormalities in VIP-containing nerves in colonic circular smooth muscle^45^. Idiopathic chronic constipation can correlate with neural abnormalities that consist of a reduced number of myoenteric plexus neurons and a decreased concentration of VIP-positive nerve fibers within circular muscle^46^.

Other studies found the opposite results: VIP expression was significantly increased in the blood, colonic mucosa, and sigmoid colonic mucosa of patients with IBS-C and IBS-D compared with the control group^47^. However, chronic constipation could be attributed to abnormal peptidergic innervation of the colonic enteric nervous system, which may manifest as a reduction in VIP levels. Moreover, VIP deficiency, recognized by the gut microbiota, leads to dysregulated proliferation, migration, and maturation of colon crypt cells, as well as dysregulated bioactive goblet cell peptide secretion, and thus unbalanced tissue repair and homeostasis. VIP also affects intestinal barrier function and is involved in the transepithelial passage of live bacteria^48^. The composition and function of the microbiota are essential for intestinal homeostasis and affect the functioning of the central nervous system. Cholinergic system, vagal afferent, and enteric nervous system (ENS) mediators such as VIP may therefore play key roles in the pathogenesis of dysbiosis and lead to intestinal permeability in constipated IBS patients.

*Ghrelin***,** an orexigenic hormone, regulates growth hormone secretion, gastric acid secretion and gastrointestinal motility^49^. It has been shown that the density of ghrelin cells in the oxyntic mucosa of the stomach is lower in IBS-C and higher in IBS-D patients than in healthy controls^50^. Decreased basal and postprandial ghrelin levels were observed in patients with functional dyspepsia^51^. In line with other studies, our study detected abnormally low basal preprandial ghrelin levels in IBS patients compared to healthy participants and a significant postprandial drop in ghrelin levels in both groups. A significant postprandial decrease in ghrelin levels was correlated with a shortened normogastria time on electrogastrography.

Ghrelin modulates the brain–gut axis and suppresses the afferent discharge of the vagus nerve, whereas CCK stimulates it. Ghrelin activates gastrointestinal motility, induces the migrating motor complex and accelerates gastric emptying^52^. Abnormalities in the low secretion of ghrelin and CCK in our patients may be the cause of gastrointestinal motility dysfunction. The main symptoms are slow transit and constipation. It is hypothesized that ghrelin may interact with motilin in IBS patients and, along with low vagal activity, contribute to the characteristic dysmotility found in IBS^53^.

The peripheral secretion of ghrelin is actively regulated by cephalic mechanisms, confirmed by feeding behavior and many metabolic reactions in anticipation of scheduled meals^54^. Since most of these conditional responses (e.g., salivation, gastric motility and acid secretion, and insulin secretion) are mediated by the ANS, the low preprandial ghrelin surges and high insulin levels in IBS patients in our study may be due to impaired autonomic control of the enteric intestinal system. Ghrelin affects the exocrine and endocrine function of the pancreas and glucose metabolism. Ghrelin worsens glucose tolerance by reducing insulin sensitivity, which is associated with a lower rate of glucose utilization and a higher rate of glucose production in the liver^55^.

*Insulin* is a physiological and dynamic modulator of ghrelin plasma concentration^56^*.* In our study, IBS patients showed significantly higher insulin levels preprandially and postprandially. Studies on the interactions among plasma insulin, glucose, and ghrelin and the effect of ghrelin on insulin secretion have shown both a stimulating and an inhibitory effect^57^. Insulin can suppress plasma ghrelin regardless of the glucose level, and it is hypothesized that a low ghrelin concentration may be a risk factor for insulin resistance^58^. Additionally, based on our EGG analysis, we noted that a high insulin level may indirectly affect gastric myoelectric activity through changes in ghrelin concentration.

*Cholecystokinin* (CCK) stimulates gastrointestinal motility, inhibits gastric emptying, and influences the secretion of pancreatic enzymes and gallbladder contraction. CCK is involved in the sensory and motor responses to gastrointestinal distension and increases the sensitivity of the rectum to balloon distension in healthy individuals^59^. These effects contribute to the constipation, flatulence and abdominal pain characteristic of functional gastrointestinal disorders. The reported evidence for CCK irregularities in IBS is not conclusive. In our study, preprandial CCK levels were significantly lower in IBS patients than in the control group. In contrast, in another publication, colonic responses to CCK in vivo and in vitro were increased in IBS patients^11,60^. The effects of CCK are mediated by two distinct receptors, CCK1 and CCK2, which are located predominantly in the periphery and the CNS. The densities of duodenal secretin and cholecystokinin cells are decreased in IBS-D patients but unaltered in IBS-C patients^61^. The motor effects of CCK include postprandial inhibition of gastric emptying and inhibition of colon transit. In our study, the baseline preprandial CCK plasma concentration was very low, and it increased by almost fourfold after a meal. In the control group, this concentration only increased by 0.2-fold compared to the baseline value.

The increased CCK release after a meal may well be involved in the exaggerated postprandial colonic motor response in IBS patients^62^ and appears to be abnormally prolonged. It has been postulated that CCK sensitizes gut mechanoreceptors and increases rectal sensitivity to pain in healthy participants, possibly through the activation of tension receptors^59^. Intraduodenal infusion of lipids increases visceral sensitivity in both IBS-C and IBS-D in the postprandial period^63^.

Abnormalities in the secretion of selected hormones in IBS patients affect the enteric nervous system, which affects bidirectional communication between the brain and the gut. The abnormal brain–gut interaction explains the pathophysiology of the body’s complex neurobiological response to stress through reactions of the hypothalamic–pituitary–adrenal axis and the hypothalamus-autonomic nervous system axis. The sympathovagal imbalance of the ANS may be associated with increased hypersensitivity to visceral pain. Alterations at any neural system level can affect motility, secretion, immune function, and perception and emotional response to visceral stimuli in the gastrointestinal tract. A variety of physiological and psychological factors are related to sex differences. Women with IBS experience more significant fatigue, depression, anxiety and lower quality of life than men. It has been observed that female sex and anxiety predict a high degree of eating-related symptoms in IBS^64^.

Most IBS patients consider and associate their symptoms with being related to meals. Foods that are high in carbohydrates and fats in particular cause problems. Nevertheless, most IBS patients are of normal weight or are overweight. Rectal barostat testing showed a higher postprandial intensity of urge and pain in IBS-C and IBS-D patients^65^. The exaggerated sensory component of the gastrocolonic reflex explains postprandial symptom onset in IBS patients^18^. The assessment of ileocolonic motor response by scintigraphic methods showed impaired ileocolonic transit in IBS-C patients^66^.

An alerted rectosigmoid response to meals suggests abnormalities in the neural pathways characteristic of functional disease. Detecting this with a barostatic device or scintigraphic method may be the pathophysiological basis for diagnosing and confirming IBS. The diagnostic value of the rectal distension test is questionable in clinical practice due to differences in the protocol used regarding the position of the balloon in the anorectum and transverse, descending, or sigmoid colon^67^.

Abnormalities in the release of hormones and peptides in the gastrointestinal tract, as discussed above, cause motility dysfunction and aggravate disease symptoms. It is important to identify specific gastrointestinal tract motor disruptions and use this knowledge in the diagnostic process. In the present study, we proposed a practical, convenient and noninvasive method for the detection of abnormal motor patterns within the gastrointestinal tract, and we focused on gastric myoelectric activity, by which we can predict intestinal abnormalities based on gastrointestinal reflexes. GMA is generated by interstitial cells of Cajal and is influenced by the central and autonomic nervous systems, as well as gastrointestinal hormones and peptides^68^. Tachygastria is an ectopic rhythm and originates from the antrum of the stomach. In contrast, bradygastria originates from the region of the normal pacemaker at a reduced frequency, is initiated in the corpus and propagates distally toward the pylorus^14^.

In the analyzed electrogastrography recordings in IBS patients, we observed a reduction in normogastria, a significant increase in dysrhythmias and a decreased percentage of slow-wave propagation and coupling, all of which indicate low gastric contractility. In our study, IBS-C patients had gastric hypomotility and uncoordinated gastric contractions correlated with dysrhythmias, irregular gastric slow-wave propagation, and an insufficient postprandial APSWC amplitude increase. GMA manages the frequency and direction of stomach propulsion. Contractions do not occur during tachygastric or bradygastric periods; therefore, tachygastria may be associated with gastric hypomotility^51,52^. Our study observed a higher percentage of tachygastria in IBS patients than in healthy controls, which is usually associated with delayed gastric emptying. Moreover, the power of EGG in IBS patients decreased significantly in the postprandial state, while in healthy controls, it increased, which is the expected physiological response after feeding. A comparison of the postprandial to fasting power ratios between the control group and IBS patients showed how severely gastric contractility is impaired in IBS patients.

The present results support the use of EGG in clinical practice as a valuable tool for the discrimination of functional dysfunction in patients with IBS. With proper standardization of procedures and reference values for some parameters, EGG may be an essential and irreplaceable test in the diagnosis and follow-up of IBS patients with gut motor dysfunction. EGG has not been widely adopted clinically due to several technical limitations, but this study may be a prelude to the use of a new method that reduces the classic EGG restraints^36^. It is a noninvasive body surface gastric mapping (BSGM) method that overcomes these limitations^69–71^.

Some previous studies have already investigated the relationship between the lack of increased postprandial EGG amplitude and delayed gastric emptying in a subset of IBS patients^72^ and delayed intestinal transit in constipation-predominant IBS^73^. Gastric emptying, colonic intestinal motility response, and gastric peptide release are dependent on many different variables. Further research is required to determine how the regulation of these variables would provide an opportunity to improve the clinical evaluation of IBS, make better use of new therapeutic approaches (e.g., the modulation of the enteric nervous system by the micropolarization technique of transcranial direct current stimulation (tDCS)), and make more appropriate recommendations for diet modification and the regulation of the intestinal microbiota.

Pharmacological therapy with certain hormones of the brain–gut axis is considered a novel treatment for FGIDs. In particular, ghrelin, which exhibits a variety of bioactivities, has attracted attention as a novel therapeutic drug^74^.

In conclusion, disturbances in the plasma levels of gut peptides may contribute to abnormal gastric activity and subsequently intestinal motility, as manifested by the clinical symptoms of IBS patients, such as the presence of visceral hypersensitivity or a predominant bowel habit. Disturbances in postprandial gastrointestinal peptide secretion (gastrin, insulin and ghrelin) correlate with abnormal gastric myoelectric activity in these IBS patients.

No clear pattern of changes in intestinal peptide alterations that could be involved in the pathogenesis of IBS has been established. Even statistically significant results often contradict previously published data, or subsequent studies cannot replicate previous significant findings. A possible reason for this discrepancy may be the heterogeneity of IBS.

Obtaining many statistically significant data, despite occasional contradictions, encourages further research on the relationship between intestinal peptides and gastric activity, as they can serve as a basis for understanding various pathophysiological pathways.

## Data Availability

The datasets used and/or analyzed during the current study are available from the corresponding author on reasonable request.
